# Hydrogel Based on Decellularized Bovine Trabecular Extracellular Matrix Enriched with Type I Collagen

**DOI:** 10.1021/acsomega.5c04026

**Published:** 2025-11-24

**Authors:** Marizia Trevizani, Laís Lopardi Leal, Gabriela Coelho Floriano, Maria Clara Bertorelli Mancini, Silvioney Augusto da Silva, Humberto de Mello Brandão, Breno Valentim Nogueira, Paulo Díaz-Calderón, Fabiano Freire Costa, Jair Adriano Kopke de Aguiar, Carlos Magno da Costa Maranduba

**Affiliations:** † Department of Biology, Laboratory of Human Genetics and Cell Therapy, Institute of Biological Sciences, Federal University of Juiz de Fora, Juiz de Fora 36036-900, Minas Gerais, Brazil; ‡ Brazilian Agricultural Research Corporation-Dairy Cattle, Brasília 70770-901, Minas Gerais, Brazil; § Carlos Alberto Redins Cellular Ultrastructure Laboratory (LUCCAR), Department of Morphology, Health Sciences Center, Federal University of Espírito Santo, Vitoria 29047-105, Espírito Santo, Brazil; ∥ Biopolymer Research and Engineering Laboratory (BIOPREL), School of Nutrition and Dietetics, Faculty of Medicine, Universidad de los Andes, Santiago CP7620086, Chile; ⊥ Department of Pharmaceutical Sciences, Faculty of Pharmacy, Federal University of Juiz de Fora, Juiz de Fora 36036-900, Minas Gerais, Brazil; # Department of Biochemistry, Glycoconjugate Analysis Laboratory, Institute of Biological Sciences, Federal University of Juiz de Fora, Juiz de Fora 36036-900, Minas Gerais, Brazil

## Abstract

Biomaterials are increasingly important in addressing the demand for biomimetic, biocompatible, and biodegradable materials for tissue replacement, treatment, or coexistence. Biocomposites enhance chemical and mechanical properties, supporting biological integration and mechanical stability at implantation sites. This study aimed to develop a thermosensitive biocomposite hydrogel using a decellularized extracellular matrix (ECM) from bovine trabecular bone and type I collagen from bovine tendon. Tendon-derived collagen increased total collagen concentration, improving cross-linking. Bovine bones were fragmented, decellularized, lyophilized, and pulverized. Type I collagen was extracted from tendons via solubilization in acetic acid, salt precipitation, and dialysis. ECM digestion was conducted in 0.01 N HCl with pepsin (1:10 ratio) at 37 °C for 96 h. The final collagen concentration was set at 10 mg/mL, with ECM-to-tendon collagen at a 1:10 ratio. Gelation was induced by temperature and pH changes. The ECM-collagen solution was neutralized (pH 7.0–7.6) using 0.1 M NaOH (1/10 digestion volume) and 10× PBS (1/9 digestion volume) to form a pregel, which was incubated at 37 °C for gelation. Gelation time, analyzed by turbidimetry at 405 nm, showed completion in ∼50 min. Collagen incorporation was 96.7%, while glycosaminoglycans (GAGs) incorporation reached 109%. Scanning electron microscopy (SEM) confirmed a porous, reticulated structure. These results indicate the successful incorporation of bone ECM components, thermosensitivity, and potential for Tissue Engineering and Regenerative Medicine applications in bone repair.

## Graphical abstract

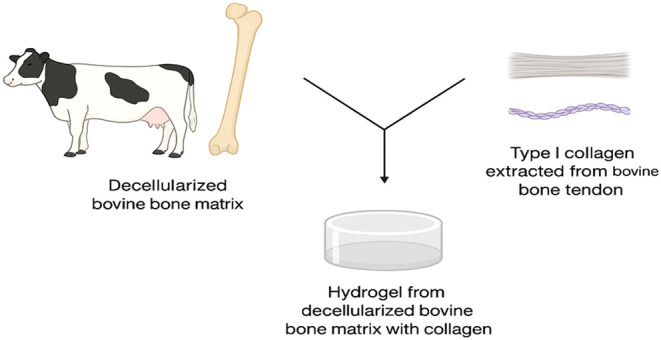

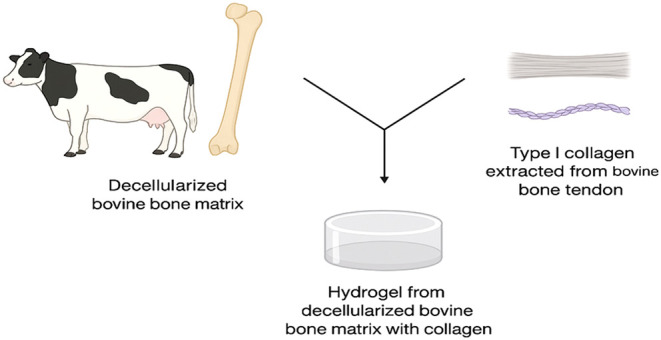

## Introduction

Bone injuries have become increasingly prevalent, driven by factors such as trauma, degenerative diseases, and the aging population, posing a significant burden on healthcare systems worldwide.^1,2^ Conventional treatments often rely on metal implants, which, while effective, are associated with limitations including risk of infection, implant loosening, pain, and the potential need for revision surgeries.^3,4^ These drawbacks have prompted the exploration of alternative therapeutic strategies that support bone healing while minimizing complications.

Due to the limited supply of allografts and the risks associated with autografts, there is growing interest in synthetic and natural bone substitutes.^5–7^ Among the natural alternatives, acellular scaffolds derived from animal bones have shown promise. When properly decellularized and sterilized, these materials are biocompatible and can act as temporary extracellular matrix (ECM) analogues, providing a three-dimensional structure that supports cell adhesion, proliferation, and differentiation, which are critical for new tissue formation.^8,9^

Decellularization has emerged as a pivotal technique in the preparation of biological scaffolds. By removing cellular components while preserving ECM architecture and bioactive molecules, the process enables host cell infiltration, immune acceptance, and functional tissue integration after implantation.^8,10^

In recent years, hydrogels have gained attention for bone tissue engineering due to their high water content, biocompatibility, and structural similarity to the native ECM. These biomaterials create a favorable environment for tissue regeneration by supporting nutrient diffusion and mimicking the biomechanical cues of native tissues.^1,5,11^ Specifically, hydrogels derived from decellularized and demineralized bone ECM demonstrate enhanced osteoinductive capacity and mechanical integrity, making them attractive for clinical translation in bone regeneration strategies.^9,12^

Therefore, the objective of the present study was to develop and physicochemically characterize a hydrogel derived from the extracellular matrix (ECM) of decellularized bovine trabecular bone, obtained using a patented decellularization protocol developed by our research group (BR 10 2022 006190 4), with the aim of exploring its potential for future applications in bone tissue engineering.

## Materials and Methods

### Extraction of Type I Collagen from Bovine Tendon

Bovine tendons obtained from the Fripai Alimentos slaughterhouse in Juiz de Fora, Minas Gerais, Brazil, were immersed in a 1% (w/v) NaCl solution (Proquímios) for transport. Subsequently, the tendons were cleaned and washed five times with a 1% NaCl solution, minced, and stored at −80 °C. Type I collagen was extracted from the bovine tendons overnight by agitation in 0.5 M acetic acid (1:8 w/v). The material was then subjected to filtration and purification following the protocol described by Chandrakasan et al.,^7^ with modifications.

In brief, the tendon/acetic acid mixture was centrifuged at 5000*g* for 30 min. Solid NaCl was then gradually added to the supernatant under constant stirring until reaching a concentration of 25% (w/v). The solution was stirred for 3 h and precipitated for another 3 h at 4 °C, followed by another centrifugation step at 5000*g* for 30 min. The resulting pellet was washed three times with 3.5% NaCl solution by centrifugation (5000*g* for 30 min) and resuspended in 0.5 M acetic acid at a volume ratio 3:8 compared to the initial extraction volume. This solution was stirred for 4 h and dialyzed against 0.5 M acetic acid for 24 h, with one buffer exchange, followed by centrifugation at 5000*g* for 30 min.

Type I collagen was precipitated from the supernatant by slowly adding, under stirring, 1/5 of the volume of 30% NaCl (w/v) in 0.5 M acetic acid over one to 2 h and then allowed to precipitate for 30 to 60 min. The precipitate was collected by centrifugation at 5000*g* for 30 min, washed once with 5% NaCl (w/v) in 0.5 M acetic acid, and resuspended in two parts of 0.5 M acetic acid. The solution was dialyzed overnight against 0.5 M acetic acid, centrifuged at 5000*g* for 30 min, and subsequently dialyzed against 0.02 M Na_2_HPO_4_ for 48 h, with multiple buffer exchanges. The precipitate was centrifuged at 5000*g* for 30 min, washed once with 0.02 M Na_2_HPO_4_, and dissolved in two parts of 0.05 M acetic acid under stirring, followed by dialysis against 0.5 M acetic acid for 24 h. Finally, the solution was aliquoted and stored at −80 °C.

### Biocomposite Hydrogel Composed of Type I Collagen and Decellularized Bovine Trabecular Bone ECM

The final collagen concentration in the hydrogel was adjusted to 10 mg/mL. To achieve this, the proportion of ECM derived type I collagen to tendon-derived type I collagen was set at 1:10. The protocol for decellularization of bovine trabecular bone was patented (BR 1020220061904)^8^ at the National Institute of Industrial Property (INPI), and it was employed to obtain the ECM from decellularized bone,^8^ thereby facilitating biomaterial production. Based on collagen quantification of the decellularized tissues, it was determined that to obtain a final concentration of 1 mg/mL of type I collagen, it was necessary to enzymatically digest 13 mg of decellularized ECM per 1 mL of the acid solution described below.

Initially, 13 mg of decellularized ECM was digested in 1 mL of 0.01 N HCl with pepsin at a 1:10 ratio at 37 °C for 96 h or until no visible matrix fragments remained. Subsequently, 9 mg of type I collagen extracted from the bovine tendon was homogenized to the ECM solution.

The resulting biomaterial exhibits thermosensitive properties, meaning its gelation occurs due to changes in both temperature and pH. For this purpose, the ECM and type I collagen solution was neutralized (pH 7.0 to 7.6) using 0.1 M NaOH (1/10 of the digestion volume) and 10× PBS (1/9 of the digestion volume), forming what is referred to as a pregel.

The pregel was subsequently gelled at 37 °C in an incubator (Model RCO3000TVBB, REVCO Technologies, Asheville).

A type I collagen hydrogel (10 mg/mL) without decellularized bone ECM was prepared using the same procedure described above and served as a control.

### Turbidimetry

The total gelation time of the biomaterials was determined by turbidimetric analysis (gelation kinetics) of each developed hydrogel, with or without decellularized bone ECM, following the methodology described by Sawkins et al.^9^

For this purpose, 100 μL of the pregel solutions were maintained at 4 °C and transferred to precooled 96-well plates. The plates were then placed in a preheated microplate spectrophotometer reader (Thermo Scientific Varioskan Flash) at 37 °C, and the turbidity of each well was measured at 405 nm every 3 min for a total duration of 1 h and 30 min. The absorbance values for each well were recorded.

Six individual measurements (*n* = 6) of each hydrogel type were performed, and the absorbance results were normalized on a scale from 0 (at time 0) to 1 (at maximum absorbance), as represented by the equation below:

$$NA=\frac{A-A_{0}}{A_{\max}-A_{0}}$$

where *A* is the absorbance at a given time; *A*_0_ is the initial absorbance; *A*_max_ is the maximum absorbance, and NA is the normalized absorbance.

### Percentage of GAGs and Collagen Incorporation in the Biomaterials

The samples were digested with papain to determine the incorporation of decellularized bone ECM into the biomaterials. Subsequently, the samples were vortexed and centrifuged at 10,000 rpm for 10 min to form a pellet containing polymer fragments. The supernatant was then used to quantify the GAGs and collagen content.

### Collagen Quantification

The collagen content in each experimental group from both protocols was determined by hydroxyproline analysis, following a modified method based on Osago et al.^10^ In this method, 10 mg of the sample (dry weight) was hydrolyzed in 1 mL of 9 N HCl (v/v) at 100 °C for 15 h to release hydroxyproline. Subsequently, 10 μL of the hydrolyzed sample was mixed with 200 μL of chloramine-T oxidizing solution [chloramine-T (Neon) 1.4% (w/v), isopropanol 10% (v/v), and sodium acetate (Proquimio) 0.5 M (w/v)] and left at room temperature for 25 min. Following this, 200 μL of Ehrlich’s reagent [para-dimethylaminobenzaldehyde (p-DAB) 1 M (w/v) in 70% (v/v) isopropanol and 20% (v/v) perchloric acid (Merck)] was added, and the mixture was incubated at 65 °C for 20 min. Absorbance was measured using a spectrophotometer at 550 nm, and the collagen content was estimated based on the assumption that hydroxyproline accounts for 14.3% of total collagen (Viswanath et al.).^11^ A standard curve was generated using known hydroxyproline (Sigma) concentrations ranging from 0 to 10 μg/mL.

### GAGs Extraction

Each experimental group from both protocols was weighed (100 mg dry weight), and GAGs were extracted through proteolysis. Papain (Proquimios, Brazil) was added to each sample at a ratio of 1 mg of papain per 100 mg of sample, along with a sufficient volume of phosphate-cysteine-EDTA buffer (Vetec; Synth; Dinâmica) (pH 6.5) to cover the material. The samples were then incubated in a water bath at 50 °C for 18 h.

The following day, the materials were filtered and centrifuged (Excelsa Baby Fanen, Brazil) at 632*g* for 15 min. GAGs were precipitated by adding 1 M NaCl (w/v) (Proquimios, Brazil) and analytical-grade ethanol (P.A.). After precipitation, the samples were stored in a freezer at −20 °C for 24 h and centrifuged at 632*g* for 15 min. The precipitates were dried in a vacuum desiccator (Unividro, Brazil), dialyzed for 48 h to remove excess salts, and subsequently lyophilized (1.0 mbar, −52 °C).

### DMMB Assay for GAGs Quantification

The 1,9-dimethylmethylene blue (DMMB) method, as described by Viswanath et al.,^11^ quantifies sulfated GAGs based on the principle that the dimethylmethylene blue dye (Sigma-Aldrich) binds to sulfate groups in the molecule, generating metachromasia. For this assay, 50 μL of the sample was added to a 96-well plate along with 200 μL of DMMB solution [sodium formate (Reagen, Brazil) 0.03 M (w/v), DMMB 4.6 × 10^–5^ M (w/v), ethanol 0.5% (v/v), and formic acid (Dinâmica, Brazil) 0.2% (v/v)]. Absorbance was measured using a spectrophotometer (Thermo Scientific Multiskan GO) at 525 nm. A standard curve was constructed using known concentrations of chondroitin sulfate (Sigma) ranging from 0 to 3 μg/mL.

### SEM of Biomaterials

SEM analysis was performed following a modified protocol based on Sawkins et al.^9^ to evaluate the ultrastructure of the biomaterials. Hydrogel samples (100 μL per well) were fixed in 10% (v/v) formaldehyde and rinsed with 1× PBS, followed by dehydration through lyophilization.

After fixation, the samples were immersed in 1% (w/v) osmium tetroxide in 0.1 M sodium cacodylate buffer supplemented with 1.25% (w/v) potassium ferrocyanide. The sample container was wrapped in aluminum foil and left at room temperature for 1 h in a fume hood and the dark. Subsequently, the samples were washed once with 0.1 M sodium cacodylate buffer for 30 min and twice with Milli-Q water for 30 min each. The samples were then dehydrated through graded ethanol baths (30, 50, 70, 90, and 100%) at room temperature and dried using critical point drying (Tousimis Auto Sandri-815).

Bone fragments were then affixed onto stubs using carbon tape (EMS 77816), followed by gold sputter coating in an argon atmosphere using a metal coater (Denton Vacuum Desk V) with a current of 30 mA for 60 s, achieving an approximate coating thickness of 200 Å. The material was visualized using a scanning electron microscope (Jeol JEM6610 LV, Japan) operated at 20 kV with a tungsten filament.

### Rheology

The TA Instruments Rheometer (Discovery HR-2) was used in the experiments. The plate-to-plate geometry was used. To avoid evaporation problems, a cover was used for protection. The Peltier plate type C (Thermo Scientific) was used in all tests, being responsible for temperature control. The experiments used a flat plate geometry (diameter of 5 cm) and a gap of 0.5 mm.

The prepared hydrogels with a volume of 300 μL were carefully placed on the rheometer stage and the geometry was closed and compressed the sample very slightly (by about 15%). In the preparation, the hydrogel is in a liquid state and cross-linking was obtained at the gelation temperature.

The hydrogel of decellularized bovine extracellular ECM enriched with type I collagen was tested. The tests were performed in triplicate and the same hydrogel was used only once.

Rheological analysis of the samples was conducted to evaluate gel formation as a function of temperature (temperature scan). The gelation temperature of the hydrogels was determined by means of oscillatory tests using a heating ramp from 15 to 50 °C at a controlled rate of 1 °C/min. The test conditions were set at a constant frequency of 1 Hz and a small deformation of 0.01% to minimize any disturbance in gel formation and simulate physiological conditions.(29) The evolution of the elastic modulus (*G*′), viscous modulus (*G*″) and tan delta (*G*″/*G*′) was monitored during the heating ramp.^12^

A viscosity versus force test was also performed on the decellularized bovine bone matrix hydrogel.

Data acquisition and analysis were performed using TRIOS software for test execution and TRIOS software for subsequent analysis.

### Cell Culture of Stem Cells from Human Deciduous Dental Pulp (SHED)

Isolation of SHEDs was performed after approval by the Human Research Ethics Committee of the Federal University of Juiz de Fora (Protocol CEP-UFJF 2263.003.2011 Report 03/2011 CAEE234.0.180.00–10).

The isolated SHEDs were thawed and cultured (5.4 × 10^6^ cells/mL) in MEM medium (Gibco) supplemented with 1% antibiotics, 1% nonessential amino acids, 1% l-glutamine, and 10% FCS in 96-well plates. Cultivation was carried out in an incubator (incubator model REVCO3000 Technologies, Asheville) at 37 °C, 5% CO_2_ in atmospheric air, and 95% humidity. The SHEDs (70% confluent) were enzymatically disaggregated by trypsin for approximately 3 min to detach them from the bottom of the bottle. Then, trypsin was inactivated with DMEM medium added with 10% (v/v) FBS, and the suspension was centrifuged (Spectrafuge 6C, Lab net Internacional Inc.) in a falcon tube at 252 G for 5 min. The supernatant was discarded and the pellet resuspended in 1 mL of supplemented DMEM medium. The cell number was determined using the Neubauer chamber.

### Cell Proliferation Analysis by Thiazolyl Blue Tetrazolium Blue (MTT)

In two 96-well cell culture plates, 100 μL of the hydrogel (prepared as previously mentioned) was added. The hydrogel was placed in the incubator for gelation. After complete gelation of the hydrogel, 200 μL of DMEM and 1 μL of the diluted cells per well were added to perform the 3- and 5-day analyses. Wells with only stem cells were used as positive control.

To assess cell proliferation, the MTT assay was used, following the manufacturer’s protocol. Initially (Day 0), 500 cells were plated per well in a 96-well plate (CORNING), and readings were taken on days 3 and 5. The culture medium was removed, and 90 μL of culture medium supplemented with 10 μL of MTT was added. The plate was then kept in the dark in an incubator at 37 °C for 4 h. After this period, the medium supplemented with MTT was removed and 100 μL of acidic isopropyl alcohol was added. The plate was kept in the dark in an incubator at 37 °C for 1 h. The supernatant was transferred to a new plate, and 100 μL of acidic isopropyl alcohol was added as a control. Proliferation was assessed in a spectrophotometer (Thermo Scientific Varioskan Flash) at absorbances of 570, 650, and 690 nm. Experiments were performed in triplicate.

### Statistical Analysis

The results from the evaluation of biomaterial development protocols are presented as mean ± standard error of the mean (SEM), unless otherwise indicated. Data from the experimental groups were analyzed using a nonparametric Student’s *t* test (Mann–Whitney test). Differences were considered statistically significant when *p* < 0.05. All statistical analyses were performed using Prism 8.0 software (GraphPad Software, Inc.).

## Results

### Turbidimetry

The hydrogel, composed of type I collagen extracted from bovine tendon and digested in HCl, takes approximately 55 min to undergo gelation, the minimum plateau time of gelation kinetics (Figure 1). In contrast, the hydrogel derived from decellularized bone ECM, enriched with Type I Collagen extracted from tendon and digested in HCl, requires approximately 50 min for complete gelation (Figure 1).

**Figure 1:**
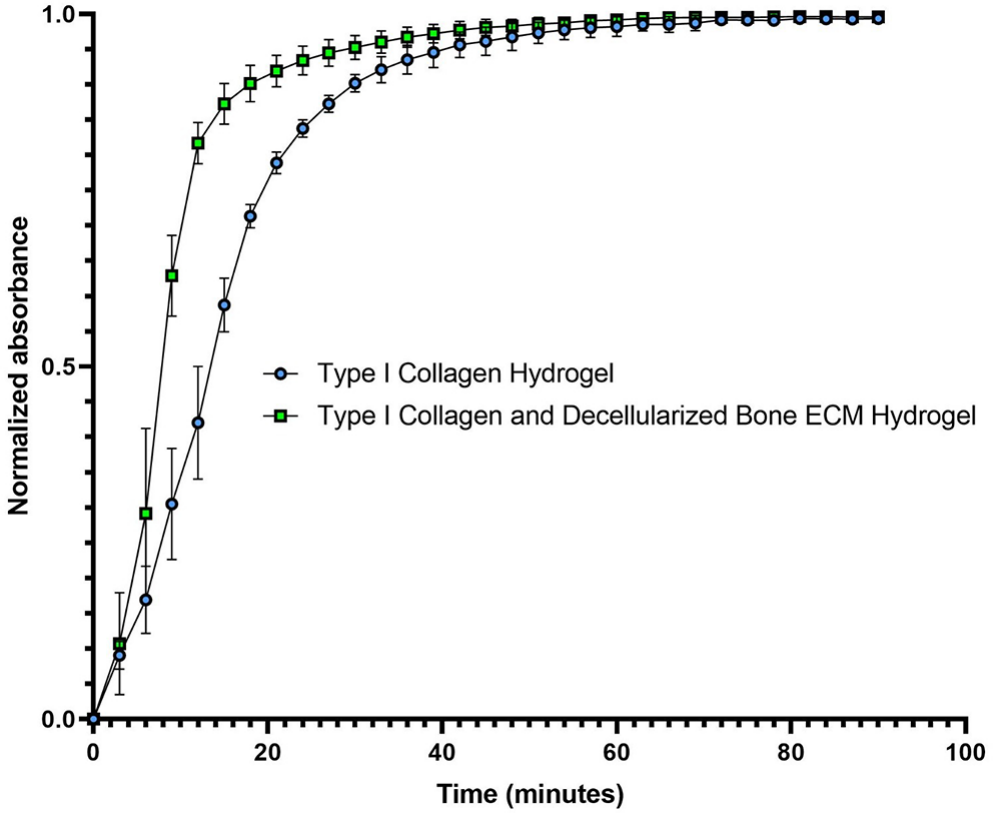
Gelation Kinetics of the Hydrogels. The experiment was performed in triplicate.

### Macroscopic Analysis of the Hydrogels

After gelation, the hydrogels were macroscopically analyzed through photography, ruler-based measurements, and opacity assessment. Differences in the hydrogel preparation revealed differences in their physical structure, including shape and opacity. The hydrogel prepared only with type I collagen did not present such a uniform shape (shape of the well of the 96-well plate) and its medium was more transparent, which shows a lower level of cross-linking in this region. The hydrogel prepared with decellularized ECM and enriched with type I collagen presents a more homogeneous shape and cross-linking (Figure 2).

**Figure 2:**
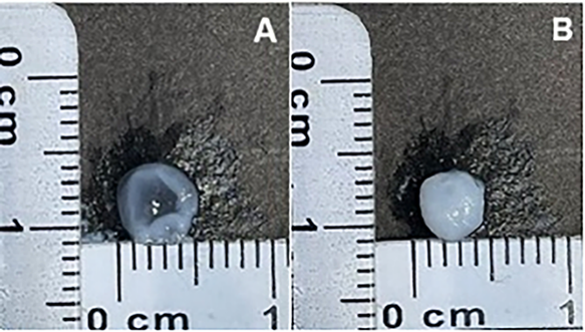
Macroscopic Analysis of the Hydrogels.. (A) Hydrogel composed of collagen I extracted from the bovine tendon. (B) Hydrogel derived from decellularized bovine bone ECM enriched with collagen type I extracted from bovine tendon. Photograph courtesy of Gabriela Coelho Floriano Copyright 2025.

### Percentage of GAGs and Collagen Incorporation in Biomaterials

The percentage of GAGs and collagen incorporation into the hydrogel, both in the type I collagen hydrogel and in the type I collagen hydrogel with decellularized bovine bone ECM, was calculated by the ratio of the mass of the macromolecule in the hydrogel to the mass of the macromolecule in the pregel, i.e., (mass of GAG in the hydrogel/mass of GAG in the pregel) × 100%. The pregel constitutes the solution of type I collagen or type I collagen with decellularized bovine bone ECM before gelation was induced by neutralization and temperature change (Figure 3).

**Figure 3:**
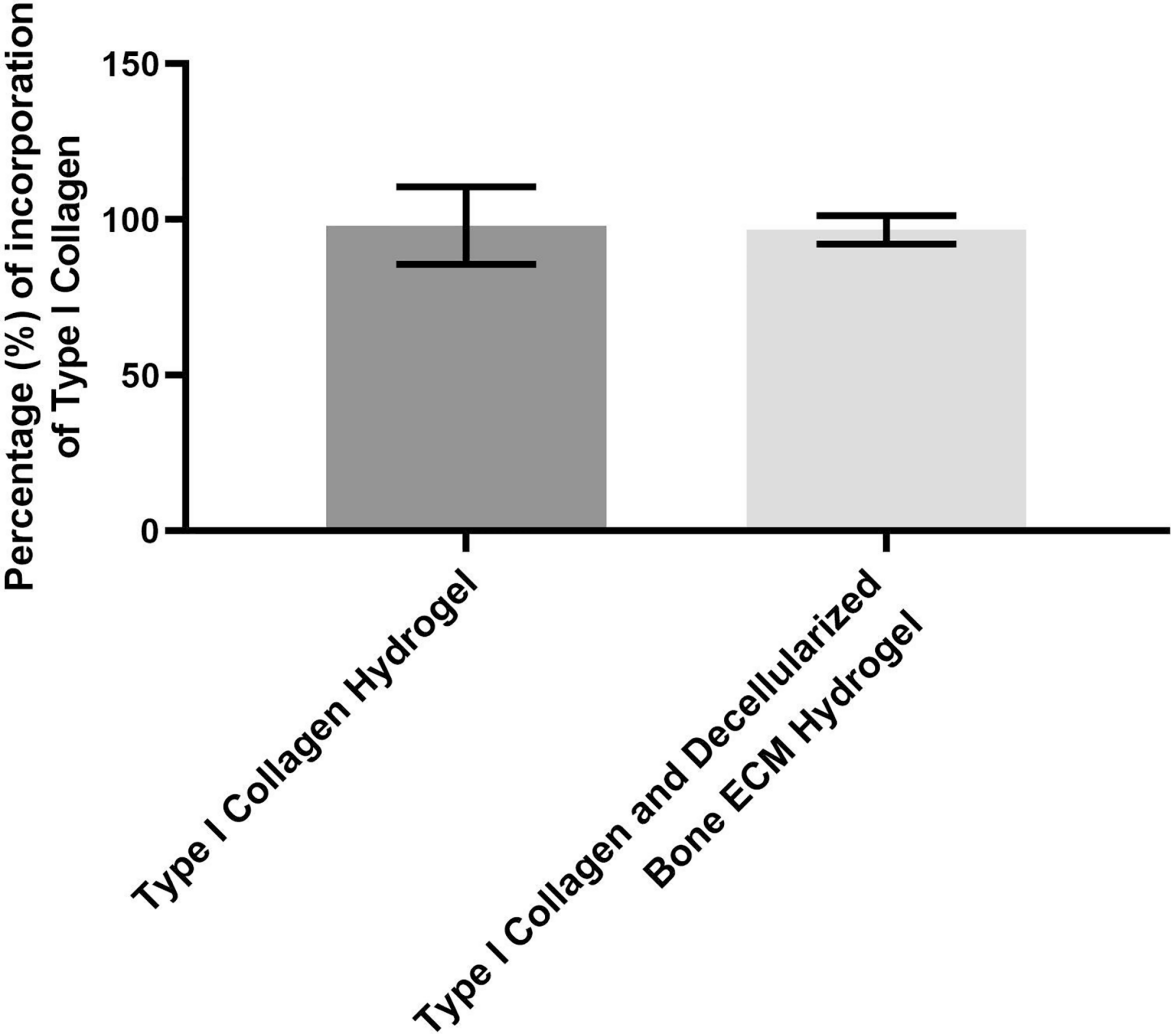
Incorporation of Type I Collagen in the Hydrogel Composed of Type I Collagen Extracted from Bovine Tendon and in the Hydrogel Containing Decellularized Bovine Bone ECM with Type I Collagen Extracted from Bovine Tendon. The experiment was performed in triplicate.

The incorporation of GAGs in the hydrogels is presented in Figure 4. In the hydrogel composed solely of type I collagen extracted from the bovine tendon, no GAGs incorporation was observed. However, for the hydrogel containing type I collagen extracted from the bovine tendon combined with decellularized ECM, the GAGs incorporation rate was 109% (Figure 4). In this case, the incorporation of GAGs above 100% in the type I collagen hydrogel with decellularized bovine bone ECM can be justified by the interference of polyanions (such as DNA, RNA and high salt concentrations) to which the DMMB method is subject.^13,14^ Thus, the addition of the neutralization and buffering solution may have overestimated the quantification of GAGs in the type I collagen hydrogel with decellularized bovine bone ECM.

**Figure 4:**
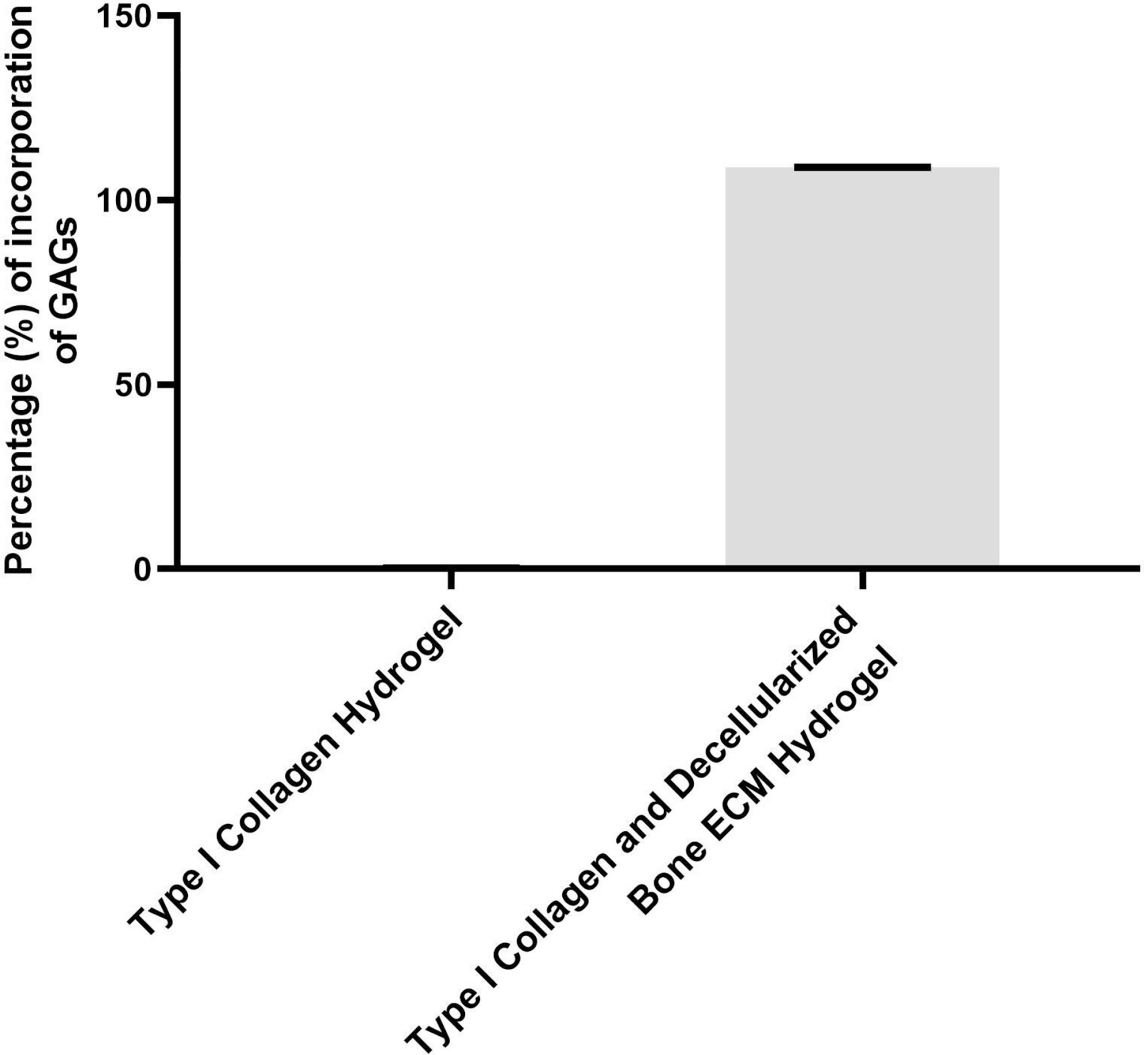
Incorporation of GAGs in the Hydrogel Composed of Type I Collagen Extracted from Bovine Tendon and in the Hydrogel Containing Decellularized Bovine Bone ECM with Type I Collagen Extracted from Bovine Tendon.

### Microstructural Analysis of Hydrogels by SEM

The images obtained through SEM (Figure 5) revealed that the hydrogels exhibit a porous structure, though their morphology varies according to the formulation. When comparing the type I collagen hydrogel extracted from the bovine tendon with the hydrogel containing decellularized bovine bone ECM with Type I collagen, both display a porous structure; however, the latter exhibits a denser structure at lower magnification. This observation may highlight the role of ECM in the gelation process. Additionally, network structures can be observed, indicating the cross-linking of collagen fibers.

**Figure 5:**
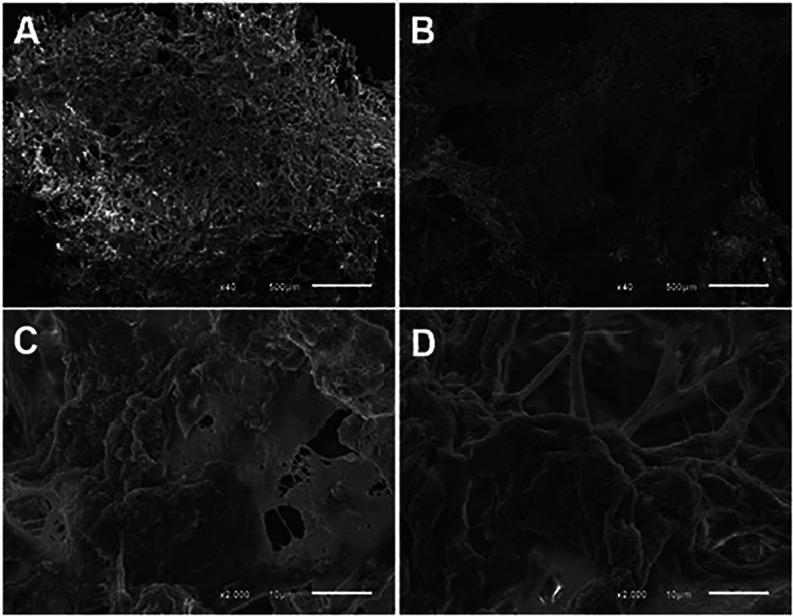
SEM images of hydrogels composed of Type I collagen extracted from the bovine tendon (A and C) and hydrogels containing decellularized bovine bone ECM with Type I collagen extracted from the bovine tendon (B and D).

Stimulating gelation directly in the rheometer proved to be a good alternative, as the equipment offers more controlled conditions and the hydrogel is temperature-sensitive. Based on the rheological measurements, it can be inferred that the bovine bone hydrogel cross-links at 37 °C and has a hardness of approximately 3 Pa. The hydrogel can be classified as non-Newtonian Herschel-Bulkley, meaning that the greater the force applied to the hydrogel, the lower its viscosity (Figures 6 and 7).

**Figure 6:**
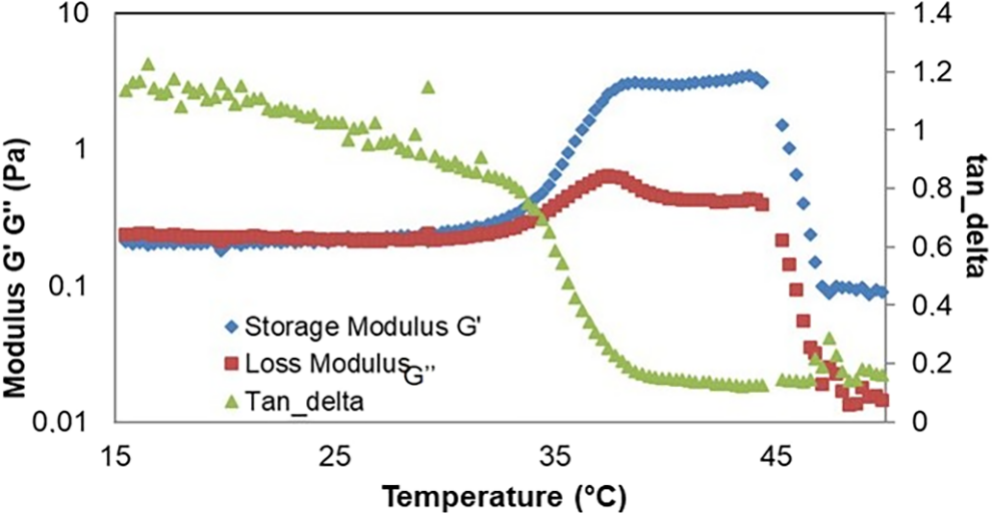
Rheology of decellularized bovine bone matrix hydrogel.

**Figure 7:**
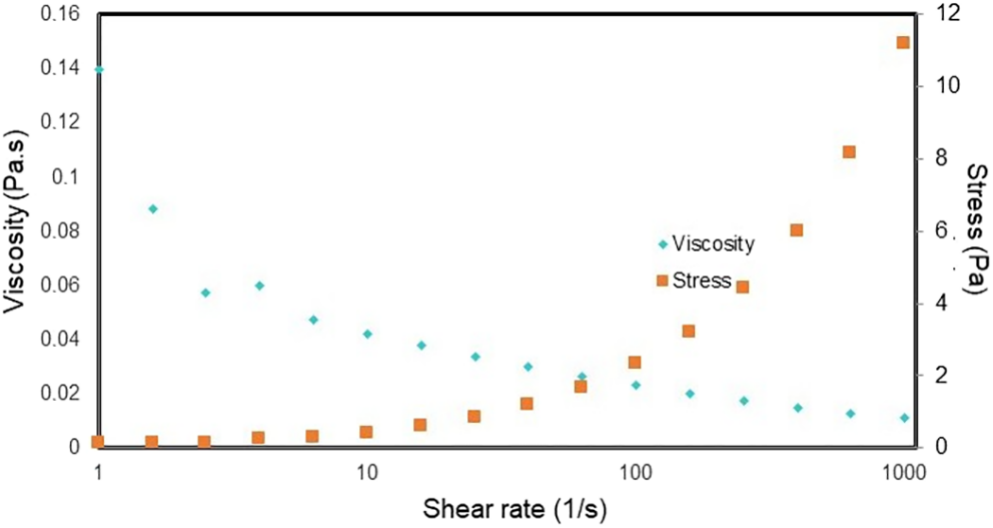
Viscosity versus force applied to decellularized bovine bone matrix hydrogel.

### Rheology

The viscoelastic behavior of the samples was evaluated by assessing the elastic modulus (*G*′) and viscous modulus (*G*″) over a range of temperatures. Figure 6 shows the representative curves of the hydrogels. The results indicate that, at all temperatures tested, the elastic behavior predominates over the viscous behavior (*G*′ > *G*″), confirming the formation of the gel.

The elastic modulus (*G*′) reaches its maximum value at temperatures close to 37 °C, in line with the theoretical temperature of gel formation. In addition, it was observed that the hydrogels composed of decellularized bovine bone ECM combined with type I collagen extracted from bovine tendon present *G*′ values close to 3 Pa. The hydrogel can be classified as non-Newtonian Herschel-Bulkley, that is, the greater the force applied to the hydrogel, the lower its viscosity (Figure 7).

### MTT Analysis

The MTT cell viability assay demonstrated differences between the groups over time. The positive control presented the highest absorbance values on days 3 and 5, demonstrating greater metabolic activity. The type I collagen hydrogel showed intermediate values, with a significant increase between days 3 and 5, indicating cell proliferation. The collagen hydrogel combined with decellularized bone extracellular matrix presented the lowest absorbance values in both analyzed periods (Figure 8). However, a significant increase was also observed between days 3 and 5, suggesting that the material does not have a cytotoxic effect. Considering that the hydrogel containing decellularized bone ECM has less rigidity compared to pure collagen, this factor may favor cell adhesion and proliferation in the collagen hydrogel, explaining the superior results observed in this group. Nevertheless, the increased cellular activity in the hydrogel with ECM demonstrates the material’s potential for application, as it allows for cell viability and growth, albeit at a lower intensity.

**Figure 8:**
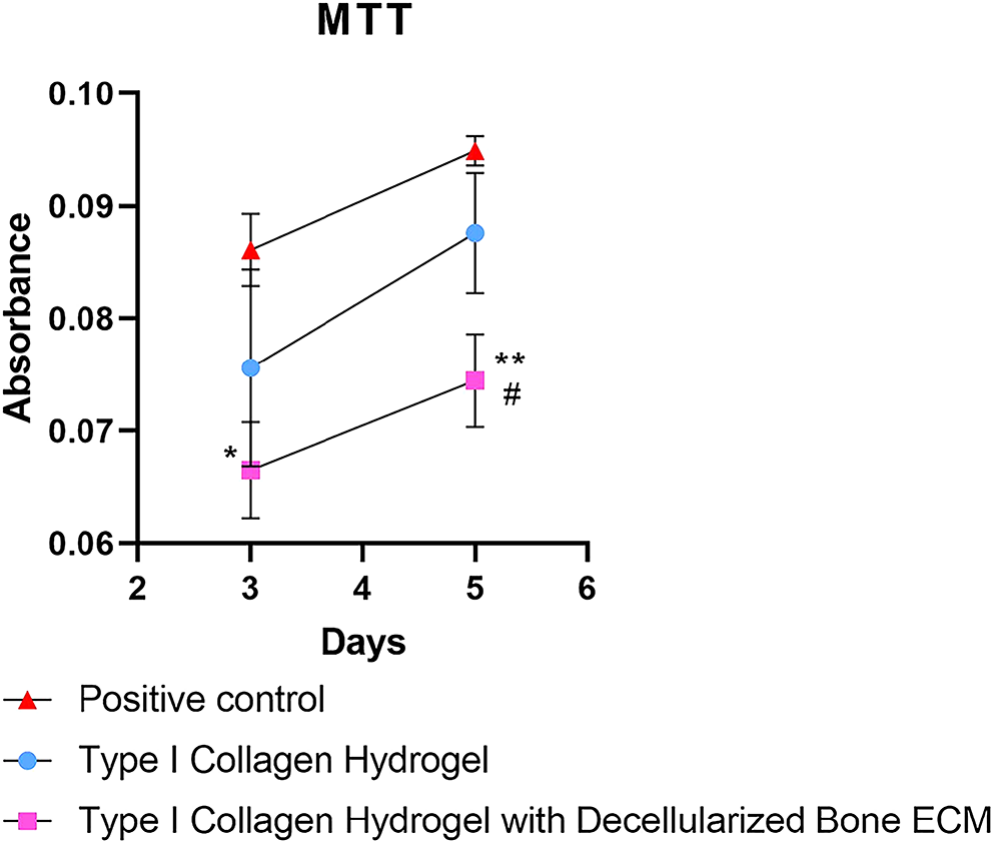
Cell viability assessed by the MTT assay under different culture conditions. The positive control showed the highest metabolic activity, followed by the type I collagen hydrogel. The collagen hydrogel associated with decellularized bone extracellular matrix showed lower values, but with a significant increase between days 3 and 5, indicating the absence of cytotoxicity. Symbols indicate statistical differences between groups (*p < 0.05; **p < 0.01; #*p* < 0.05 compared to type I collagen).

## Discussion

Tissue engineering has emerged as a promising alternative for the repair of bone injuries, with hydrogels being widely used as scaffolds due to their three-dimensional hydrophilic structure and ability to mimic the natural extracellular microenvironment.^5,15^ These biomaterials allow the transport of drugs, extracellular vesicles, and stem cells, promoting adhesion, cell differentiation, and tissue regeneration.^16–19^

The choice of cross-linking agent is crucial in defining the physicochemical and biological properties of the hydrogel. In the present study, the incorporation of type I collagen, extracted from bovine tendon, accelerated gelation at 37 °C, favoring its injectable application and molding at the site of the injury, with an incorporation efficiency of 96.7%. The comparison with the control (hydrogel with collagen from tendon only, with 98.1% incorporation) indicates that the decellularized extracellular matrix (ECM) does not interfere in the process at any level. Unlike alginate-based ionic hydrogels cross-linked by CaCl_2_, which exhibit immediate and heterogeneous gelation,^20^ the hydrogel developed exhibits thermoinduced gelation without the need for toxic or cytotoxic chemical cross-linking agents.^20–22^ This characteristic, combined with the possibility of injectable administration, represents an advance over traditional surgical approaches, which are invasive and require prolonged recovery.^23^

Type I collagen extracted from bovine tendon was quantified, according to Osago et al.,^10^ to calculate the purity of the extraction, identified by SDS-polyacrylamide gel electrophoresis (SDS-PAGE). Thus, the purity was approximately 37.93 ± 7.36%, and the extracted compound was, in fact, type I collagen, since it presented electrophoretic bands specific for the monomer, dimer, and trimer of the molecule.

Type I collagen is widely used as a structural component and natural cross-linking agent, and is essential for the stability and bioactivity of scaffolds.^24–26^ Its high hydrophilicity contributes to water retention, while its interaction with other ECM components, such as GAGs, reinforces the polymer network.^27,28^ In the present study, the GAGs incorporated into the hydrogel showed incorporation rates of 109%, evidencing the maintenance of ECM macromolecules during the decellularization and formulation processes. The absence of GAGs in the control group (0%) corroborates the exclusive contribution of bone ECM to this incorporation.^29–31^

A scanning electron microscopy (SEM) analysis revealed porous structures in both hydrogels tested, but the ECM derivatives exhibited greater density and more pore distribution. This indicates that the ECM contributes to a microstructure that is more favorable to cellular resistance and mechanical resistance.^28,32^

Rheology demonstrated that the hydrogel presents viscoelastic behavior compatible with pseudoplastic materials, that is, its characteristics decrease with increasing shear rate. This profile was associated with the presence of GAGs, which directly affect the mechanical properties of the polymer network.^9^ The elastic modulus *G*′ was approximately 3 Pa after gelation, and the structure broke completely at temperatures above 45 °C, reducing thermal sensitivity. The compliance curve showed three zones: an initial one with chain alignment, a Newtonian plateau between 2.5–4 s^–1^ and, subsequently, network disorganization and a decrease in consistency, a behavior similar to that described in hydrogels based on porcine bladder matrix^33^ and anionic micelles.^34^

The data demonstrate that the combination of decellularized bone ECM with type I collagen results in a hydrogel with excellent gelation profile, biocompatibility and adequate rheological properties, highlighting its potential for injectable applications in bone regeneration therapies.

The results obtained in the MTT assay demonstrate significant differences in cell viability and proliferation between the groups analyzed. The positive control group, composed only of stem cells cultured in conventional medium, showed a higher proliferation rate over time, as expected, since two-dimensional culture conditions in treated plastic favor cell adhesion and expansion.^35^

The type I collagen hydrogel also allowed stem cell adhesion and proliferation, although at slightly lower levels than the control. This result corroborates previous studies that indicate collagen is one of the main components of the extracellular matrix, capable of promoting structural support, cellular interaction through integrin receptors, and the maintenance of specific phenotypes.^36,37^ The observed proliferation suggests that the material is biocompatible and does not exhibit significant cytotoxicity, reinforcing its potential for tissue engineering applications.

On the other hand, the collagen hydrogel combined with decellularized bone extracellular matrix (ECM) showed lower absorbance values compared to the other groups, indicating reduced cell proliferation. This result may be related to several factors. First, the decellularization process can alter the biochemical composition of the ECM, leading to the loss of some bioactive factors essential for cell signaling.^38^ Furthermore, residues of chemical agents used in decellularization, if not completely removed, can compromise cell adhesion and viability.^38,39^

It is also important to consider that bone ECM has distinct characteristics from the matrix of the tissue of origin of the stem cells used. While isolated type I collagen provides a more generic support environment, decellularized bone ECM contains bone-specific proteins and glycosaminoglycans, such as osteopontin and osteocalcin, which can induce early differentiation signals to the detriment of proliferation.^40^ Thus, the lower cell growth rate may be associated with the activation of osteogenesis-related pathways, which is relevant for bone regeneration applications.

Taken together, the results indicate that type I collagen hydrogels are biocompatible and allow stem cell proliferation, albeit at a lower rate than conventional culture. The addition of bone ECM negatively modulated cell expansion, possibly directing the cells toward more specialized phenotypes. Therefore, future studies should include additional analyses, such as osteogenic marker labeling, mineralized matrix deposition, and gene expression assays, to elucidate whether ECM promotes specific, differentiating effects.

## Conclusion

Based on the results, the hydrogels derived from decellularized bovine trabecular ECM demonstrated potential applicability for bone repair. Due to their ability to undergo gelation at 37 °C, these biomaterials can be utilized in an injectable form, making them less invasive than currently available therapies. Furthermore, their physical and chemical properties were favorable for potential tissue regeneration, suggesting the formation of an optimal microenvironment. However, further evaluation of the hydrogel’s cytotoxicity and in vitro and in vivo experimentation is necessary to confirm its suitability as a biomaterial for clinical applications.
